# Performance evaluation of case definitions of type 1 diabetes for health insurance claims data in Japan

**DOI:** 10.1186/s12911-021-01422-z

**Published:** 2021-02-11

**Authors:** Tasuku Okui, Chinatsu Nojiri, Shinichiro Kimura, Kentaro Abe, Sayaka Maeno, Masae Minami, Yasutaka Maeda, Naoko Tajima, Tomoyuki Kawamura, Naoki Nakashima

**Affiliations:** 1 Medical Information Center, Kyushu University Hospital, Maidashi 3-1-1 Higashi-ku, Fukuoka City, Fukuoka Prefecture 812-8582 Japan; 2 Department of Molecular Medicine and Metabolism, Research Institute of Environmental Medicine, Nagoya University, Nagoya, Japan; 3 National Hospital Organization Kokura Medical Center, Fukuoka, Japan; 4 Sasaki Diabetes Clinic, Fukuoka, Japan; 5 Clinic Masae Minami, Fukuoka, Japan; 6 Jikei University School of Medicine, Tokyo, Japan; 7 Departmentof Pediatrics, Osaka City University, Osaka, Japan

**Keywords:** Predictive model, Type 1 diabetes, Validation study, Machine learning

## Abstract

**Background:**

No case definition of Type 1 diabetes (T1D) for the claims data has been proposed in Japan yet. This study aimed to evaluate the performance of candidate case definitions for T1D using Electronic health care records (EHR) and claims data in a University Hospital in Japan.

**Methods:**

The EHR and claims data for all the visiting patients in a University Hospital were used. As the candidate case definitions for claims data, we constructed 11 definitions by combinations of International Statistical Classification of Diseases and Related Health Problems, Tenth Revision. (ICD 10) code of T1D, the claims code of insulin needles for T1D patients, basal insulin, and syringe pump for continuous subcutaneous insulin infusion (CSII). We constructed a predictive model for T1D patients using disease names, medical practices, and medications as explanatory variables. The predictive model was applied to patients of test group (validation data), and performances of candidate case definitions were evaluated.

**Results:**

As a result of performance evaluation, the sensitivity of the confirmed disease name of T1D was 32.9 (95% CI: 28.4, 37.2), and positive predictive value (PPV) was 33.3 (95% CI: 38.0, 38.4). By using the case definition of both the confirmed diagnosis of T1D and either of the claims code of the two insulin treatment methods (i.e., syringe pump for CSII and insulin needles), PPV improved to 90.2 (95% CI: 85.2, 94.4).

**Conclusions:**

We have established a case definition with high PPV, and the case definition can be used for precisely detecting T1D patients from claims data in Japan.

## Background

Type 1 diabetes (T1D) is a chronic disease caused by the destruction of insulin-producing beta cells of the pancreas [1, 2]. T1D patients need to regularly self-monitor their plasma glucose level and self-inject insulin for all their life [2, 3], and have a higher risk of developing cardiovascular diseases than the general population [2]. Although there are some epidemiological studies on T1D patients in Japan [4, 5], studies on T1D using health insurance claims data are scarce. To assess the prevalence and clinical characteristics of patients with a disease, epidemiological studies using health insurance claims data are valuable, and these studies are common in Japan [6–8]. By using nationwide claims data, we can obtain the prevalence and prescription pattern of T1D patients. However, it is known that the name of the disease in the medical records is sometimes not detailed enough, as these are put for either inspection or prescription [9, 10], and relying solely on the International Statistical Classification of Diseases and Related Health Problems, Tenth Revision. (ICD 10) code is not an appropriate way of identifying a patient’s disease. Therefore, we need to decide the case definition for extracting the patient of the disease, and validation study of a case definition for extracting patients of a particular phenotype are often conducted for multiple diseases [11–13].

To conduct an epidemiological study on T1D patients, it is necessary to develop a case definition for T1D from the claims data. Although case definition methods for diabetes and type 2 diabetes have been proposed in some studies [14–19], proposals for T1D are scarce [20]. Although a review of the medical chart is usually conducted for deciding whether or not a given case is a true case of the target disease in the validation study, this activity is time-consuming and expensive. Moreover, when we review patients who match the case definition, only the positive predictive value (PPV) can be calculated (sensitivity of the algorithm cannot be calculated). Then, we used a newly proposed method called Phevaluator for evaluating the case definition algorithm [21]. Phevaluator is a machine-learning-based method of assessing phenotyping methods. It constructs a predictive model for the disease and calculates the predictive value of being the disease for each individual using the model. Using Phevaluator, we can calculate the performance indexes of the algorithm without reviewing the medical chart.

In this study, we aimed to construct some case definition methods of TID for claims data and evaluate the performance of them using the EHR and claims data of a University Hospital.

## Methods

### Study population

We used the data of a University Hospital in Japan from 2009 to 2019. Of those, the data from 2009 to 2014 were used as training data for constructing a predictive model for the disease, and the remaining were used as test data for evaluating performances of candidate case definitions. However, only patients who did not visit the hospital from 2009 to 2014 were used for test group (validation data) to separate patients in the training data and the test data. Electronic healthcare records (EHR) data and health insurance claims data were used in this study. EHR data were used for determining the “true” T1D patients in the medical chart review, as described below. From the claims data, data on age, sex, diseases, medications, and medical practices for all the visiting patients were used.

### Extraction of cases of T1D from EHR data

We extracted “true” T1D patients from EHR data. First, we extracted all possible TID patients from visiting patients from 2009 to 2014. All the “possible” patients are those who met one or more of the following five criteria.

① Patients who were diagnosed with TID or insulin-dependent diabetes.
② Patients who met all of the criteria (a), (b), and (c).
  - Those who were prescribed insulin treatment.
  - Those who had serum C-peptide immunoreactivity (CPR) less than 0.6 ng/ml at least once.
  - Those who had earlier been diagnosed with ketoacidosis.
③ Patients whose insulin autoantibody (anti-glutamic acid decarboxylase antibody; GAD or anti-insulinoma‐associated protein-2 antibody; IA2) was positive.
④ Patients who were introduced as definitely the patients with TID by diabetologists.
⑤ Patients whose serum CPR values were less than 0.2 ng/ml at least once.

Medical chart review was conducted against all the possible TID patients by three diabetologists, and each patient was either classified as “true” TID patients or not. Then, those who fell within the criteria were classified as TID patients.

### Phevaluator

Phevaluator is a machine-learning-based method of evaluating case definitions [21]. In this method, a predictive model is constructed in the training data for classifying the target disease patients and the other population. Then, we apply the predictive model to the patients of test group (validation data), and calculate the predictive probability of being a patient with the disease for each patient. We explain Phevaluator using the example where the number of patients in the validation data are 4 in Table 1. Let patients A and C be tested positive according to a candidate case definition, and patients B and D be tested negative according to the same case definition. From the predictive values of being a true patient or not for A and C, we calculate the cumulative probabilities of true positivity (TPs) and cumulative probabilities of false positivity (FPs). Similarly, from the predictive values of being a true patient or not for B and D, we calculate the cumulative probabilities of true negativity (TNs) and the cumulative probabilities of false negativity (FNs). Then, sensitivity, specificity, PPV, and negative predictive value (NPV) can be calculated as follows: sensitivity: $TPs/(TPs+FNs)$; specificity: $TNs/(FPs+TNs)$; PPV: $TPs/(TPs+FPs)$; NPV: $TNs/(FNs+TNs)$. We also calculated the F-score by $2\times sensitivity\times PPV/(sensitivity+PPV)$.

**Table 1:** Schematic table of the calculation method of performance indexes by Phevaluator

Patient	Positive by a case definition		Negative by a case definition	
	Predictive value of being true patient (TP)	Predictive value of being false patient (FP)	Predictive value of being true patient (FN)	Predictive value of being false patient (TN)
A	0.7	0.3		
B			0.4	0.6
C	0.9	0.1		
D			0.3	0.7
Cumulative probabilities	TPs = 1.6	FPs = 0.4	FNs = 0.7	TNs = 1.3

*TP* true positive, *FP* false positive, *TN* true negative, *FN* false negative

### Case definitions

As possible case definitions, we evaluated the performances of multiple definitions. We constructed definitions using the following four codes: ① Confirmed disease name of TID, ICD 10 code: of E10; ② Claims code of insulin needles for T1D patients: claims code of 114,010,970; ③ Claims code of basal insulin (long-acting insulin analog, intermediate-acting insulin, biphasic insulin); and ④ Claims code of syringe pump for continuous subcutaneous insulin infusion (CSII): claims codes of 114004810 and 114022010. The codes are closely associated with T1D. As concerns the method of glycemic control, there are two types of methods: multiple insulin injections and CSII [22]; we used the claims codes of both types of methods. Also, in Japan, medical remuneration points of prescription of injector needles for TID patients are set as same as those for hemophilia or other patients and are higher than those for other diseases. TID patients who self-inject insulin must have this claims code, and we used it. Basal insulin consists of a long-acting insulin analog, intermediate-acting insulin, and biphasic insulin. The list of claims codes for basal inulin is shown in the Additional file 1. As the codes associated with CSII, we used the claims code for the syringe pump for intermittent infusion. Through combinations of these four items, we tested the performance of 11 types of case definitions.

### Statistical analysis

We constructed the predictive model for classifying TID patients with non-TID patients using the claims data from 2009 to 2014, and the data on age, sex, and diseases, medications, and medical practices were used as explanatory variables. Regarding diseases, we distinguished between suspected diseases and confirmed disease and classified each of the ICD10 codes of diseases based on the first three digits of the ICD10 codes. For medications, we classified claims codes for medications by their generic names.

Regarding the outcome variable, the patients who were definitely patients of TID should be used as the cases in the evaluation by Phevaluator [21]. Therefore, from the patients who were classified as TID, we excluded patients whose pancreas were transplanted because their insulin secretion ability was probably boosted by the transplantation. We also excluded the patients who had either confirmed or suspected type 2 diabetes (T2D). Also, as concerns the controls, we needed to extract those who were definitely not patients with TID [21]. Then, the controls were selected from the patients whose medical charts were not reviewed. Also, those who had either suspected or confirmed TID were excluded. Furthermore, we randomly sampled the control patients to adjust the ratio of the cases and the controls [21].

Explanatory variables, except for age and sex, were transformed into dummy variables based on whether a patient had a code during the periods or not. However, the variables used for extracting the true patients need to be excluded from the explanatory variables [21]. Therefore, we excluded variables of confirmed disease names of T1D, T2D, and ketoacidosis from the explanatory variables. Also, we excluded variables of suspected disease names related to T1D and T2D. Moreover, claims code for test for the insulin receptor autoantibody and CPR, and insulin medications were excluded from the explanatory variables.

The gradient-boosting decision tree was used in constructing the predictive model. However, if we use all the explanatory variables, the size of the data would become very large. Therefore, we calculated in advance the relative risk of each explanatory variable to the outcome variable and used the top 500 variables for the predictive model. The area under the curve (AUC) of the predictive model was calculated by tenfold cross-validation within the training data. A predictive model of the test data was applied to each patient, and the predictive value for T1D was calculated. Also, confidence intervals of the performance indexes were calculated by bootstrap sampling. All statistical analyses were conducted using R version 3.6.3 (https://cran.r-project.org/bin/windows/base/old/3.6.3/).

## Results

**Fig. 1:**
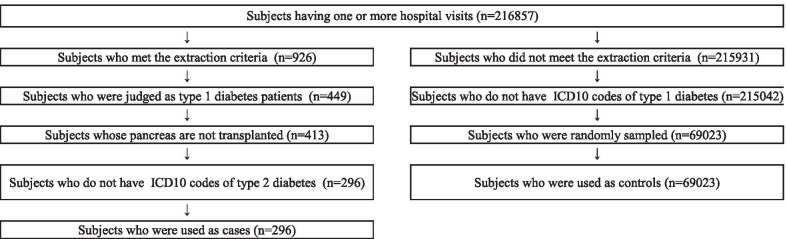
Flowchart of subjects used for the training data

Figure 1 shows the flowchart of the training data in this study. Finally, 296 patients were used as cases, and 69023 patients were used as controls.

Table 2 shows the baseline characteristics of the analyzed data. More than 7000 variables in total were used in the construction of the predictive model.

**Table 2:** Baseline characteristics of the analyzed data

	Training data	Test data
Men, n (%)	30,832 (44.5)	51,612 (45.4)
Age, mean (standard deviation)	45.8 (24.3)	44.6 (24.9)
Confirmed diseases, n	1396	1416
Suspected diseases, n	1186	1256
Medications, n	1794	1945
Medical practices, n	4017	5061

Table 3 shows the variable importance of gradient boosting decision trees, and the result of the top 10 variables are shown. The variable importance of the claims code of insulin needles for T1D patients was significantly higher than other variables, and it was suggested that the claims code is crucial for the classification of T1D patients. Also, the mean AUC of the predictive model by 10-cross validation was 0.935. Therefore, a predictive model with high classification ability was constructed using the analyzed data.

**Table 3:** Variable importance of gradient boosting decision trees (top 10 variables)

Items	Codes^a^	Importance
Insulin needles for type 1 diabetes patients	114010970	0.324
Age		0.139
Test for HbA1c	160010010	0.05
Glucometers for type 1 diabetes or child glycemia patients (more than 120 times)	160151050	0.029
Urinalysis for quantitative measurement of Albumin	160004810	0.029
Test for Glycoalbumin	114015610	0.025
Standard psychoanalytic treatment	160000310	0.019
Urinalysis	180006810	0.016
Syringe pump for intermittent infusion	114022010	0.016
Confirmed diagnosis of eating disorder	F50	0.013

^a^ICD10 Codes for diseases, and claims code for medical practices

Table 4 shows the result of the evaluation of candidate case definitions for T1D. The results of NPV and specificity are not shown in Table 4 because the values were almost 100% for all the case definitions. The sensitivity and PPV of confirmed disease T1D were relatively low. On the other hand, sensitivity and PPV for the code of insulin needles for T1D patients outperformed those of the confirmed disease, and the F-score was the highest among the candidate definitions. By combining the confirmed T1D and the claims codes of the syringe pump for CSII, PPV became the highest, but the sensitivity dropped. The 9th case definition had the highest F-score among the case definitions whose PPV was approximately 90%.

**Table 4:** The result of the evaluation of candidate case definitions for type 1 diabetes

Case definition	N (%)^e^	Performance values (95% CI)		
		Sensitivity	PPV	F-score
1. ① ICD10 code of E10^a^	305 (0.268)	32.9 (28.4, 37.2)	33.3 (28.0, 38.4)	0.331 (0.285, 0.373)
2. ② Claims code of injector needles^b^	154 (0.135)	32.3 (27.9, 36.6)	64.8 (58.9, 70.6)	0.431 (0.382, 0.475)
3. ③ Claims codes of basal insulin^c^	1392 (1.224)	40.2 (36.1, 44.5)	8.9 (7.6, 10.3)	0.146 (0.126, 0.166)
4. ④ Claims codes of syringe pump^d^	14 (0.012)	3.9 (1.9, 6.0)	86.2 (68.0, 99.0)	0.075 (0.037, 0.112)
5. ① as well as ②	73 (0.064)	21.4 (17.0, 25.4)	90.4 (85.0, 94.8)	0.346 (0.286, 0.399)
6. ① as well as ③	136 (0.120)	25.9 (21.7, 30.1)	58.8 (51.0, 66.2)	0.360 (0.307, 0.411)
7. ① as well as ④	12 (0.011)	3.6 (1.7, 5.5)	92.2 (79.0, 99.4)	0.069 (0.034, 0.103)
8. ① as well as (③ or ④)	138 (0.121)	26.4 (22.0, 30.5)	58.9 (51.0, 66.2)	0.364 (0.310, 0.414)
9. ① as well as (② or ④)	81 (0.071)	23.7 (19.5, 27.9)	90.2 (85.2, 94.4)	0.375 (0.319, 0.427)
10. ① as well as (② or ③)	136 (0.120)	25.9 (21.7, 30.1)	58.8 (51.0, 66.2)	0.360 (0.307, 0.411)
11. ① as well as (② or ③ or ④)	136 (0.120)	26.4 (22.0, 30.5)	58.9 (51.0, 66.2)	0.364 (0.310, 0.414)

*CI* confidence interval, *NPV* negative predictive value, *PPV* positive predictive value

^a^Confirmed disease name of type 1 diabetes based on ICD10 code

^b^The claims code of injector needles for type 1diabetes

^c^The claims codes of basal insulin

^d^The claims codes of syringe pump for continuous subcutaneous insulin infusion

^e^Number of cases who fell within the case definitions in the test data (prevalence within the test data)

## Discussion

We used Phevaluator for evaluating the performance of the case definitions in order to extract true TID patients from the medical records. The predictive model with high classification ability was constructed, and the performance indexes were considered to be accurately estimated. As a result of variable importance showed, the claims code of insulin needles for T1D patients was found to have the highest predictive ability. T1D patients need to inject insulin regularly [2, 3], and injection needles are considered to be vital for classifying T1D patients and non-T1D patients.

As a result of performance evaluations, PPV and sensitivity varied depending on the case definitions. Both sensitivity and PPV for the case definition of only using the ICD 10 code of T1D were not high among the case definitions for extracting the true T1D patients. It was suggested that more than half of the number of patients with T1D patients were not attached to the ICD 10 code of T1D, and there is a possibility that many T1D patients treated with other disease names except the ICD 10 code of E10. It was also demonstrated in a previous study that the sensitivity of 1 diabetes was relatively low for extracting “true” T1D patients [20]. On the other hand, the PPV increased by using the claims code of insulin needles for T1D patients, and the sensitivity was almost unchanged. It is considered that a large part of patients with confirmed diagnosis of T1D use insulin needles for T1D patients because decrease in the sensitivity was small. Furthermore, by using both the confirmed diagnosis of T1D and the claims code of insulin needles as the case definition, PPV increased further. As one possible reason, the claims code of insulin needles is used not only for T1D patients, but the same claims code is used for injector needles for hemophilia patients. Therefore, by restricting the cases to those who have ICD10 codes of T1D, PPV is considered to have improved. On the other hand, PPV did not improve by adding the code of basal insulin for the case definition. Basal insulins are required for T1D patients who cannot secrete insulin [23], and the sensitivity was highest when using only basal insulin. As the result shows, the number of extracted cases by the case definition remained almost unchanged by adding the codes of basal insulin compared with the definition of only using the insulin needles, and it is considered that patients who have the code of insulin needles tend to have a claims code of basal insulin too. Although PPV increased when using the claims codes of the syringe pump for CSII, sensitivity tended to decrease because the rate of T1D patients using CSII is lower compared with injection treatment [24]. However, the F-score of the confirmed diagnosis of TID with the claims code of either of the treatment methods has the highest F-score among the case definitions whose PPV were sufficiently large. Taking into account that a certain percentage of T1D patients use CSII in Japan [24], this case definition is considered to be useful in identifying true T1D patients.

This study has some limitations. First, we used the data of only one site for evaluating the case definitions. It is considered that some patients receive insulin medications in other clinics or hospital, and this might have affected the result. Similar validation studies need to be conducted using nationwide data for confirming the results of this study. As another limitation, although whether a candidate T1D patient is a “true” case or control was judged by diabetologists in the medical chart review for the classification of TID patients, there is still a possibility that some misdiagnosis occurred in the classification. Finally, we could not obtain a case definition whose PPV and sensitivity were both sufficiently high values, and we still need to seek claims codes for improving the sensitivity of the case definition. However, the PPV was high enough, and it was suggested that we could precisely identify true T1D patients from claims data. The actual conditions of prescription patterns, comorbidities, or medical expenditures for T1D patients is uncertain in Japan at this moment, and epidemiological studies on T1D using claims data need to be conducted by using the proposed case definition. This is the first study that derived a case definition of T1D from claims data in Japan, and further studies for case definition and epidemiological studies of T1D are needed.

## Conclusions

As a result of the performance evaluation of the case definitions for T1D, it was suggested that the ICD10 code of T1D should not be used for assessing the true patients with T1D. The F-score was highest when using both the confirmed diagnosis of T1D and either of the claims codes of two insulin treatment methods (i.e., syringe pump for CSII and insulin needles) among the case definitions whose PPV were sufficiently large. Therefore, the proposed case definition can be used for precisely detecting T1D patients from claims data in Japan.

## Supplementary information

**Additional file 1.** List of basal insulin used in the analysis.

## Data Availability

The datasets analyzed during the current study are not publicly available due hospital data were used but are available from the corresponding author on reasonable request.

## Abbreviations

ICD10: International Statistical Classification of Diseases and Related Health Problems, Tenth Revision
T1D: Type 1 diabetes
EHR: Electronic health care records
PPV: Positive predictive value
CSII: Continuous subcutaneous insulin infusion
CPR: C-peptide immunoreactivity
AUC: Area under the curve
T2D: Type 2 diabetes
