# Supplementary material for: Performance evaluation of case definitions of type 1 diabetes for health insurance claims data in Japan
Source: BMC Med Inform Decis Mak. 2021 Feb 11;21:52. doi: 10.1186/s12911-021-01422-z (PMC7879626; doi:10.1186/s12911-021-01422-z)
Supplement: Supplementary file 1 — Additional file 1. List of basal insulin used in the analysis. [file 12911_2021_1422_MOESM1_ESM.docx]

**Title:**

Performance evaluation of case definitions of type 1 diabetes for health insurance claims data in Japan

**Authors:**

Tasuku Okui^1^ (MPH, PhD)

Chinatsu Nojiri^1^ (BD)

Shinichiro Kimura^2^ (MD, PhD)

Kentaro Abe^3^ (MD, PhD)

Sayaka Maeno^4^ (MD)

Masae Minami^5^ (MD)

Yasutaka Maeda^5^ (MD, PhD)

Naoko Tajima^6^ (MD, PhD)

Tomoyuki Kawamura ^7^ (MD, PhD)

Naoki Nakashima^1^ (MD, PhD)

**Affiliations:**

1. Medical Information Center, Kyushu University Hospital, Fukuoka city, Japan.
2. Department of Molecular Medicine and Metabolism, Research Institute of Environmental Medicine, Nagoya University, Nagoya, Japan
3. National hospital organization kokura medical center, Fukuoka, Japan
4. Sasaki Diabetes Clinic, Fukuoka, Japan
5. Clinic Masae Minami, Fukuoka, Japan
6. Jikei University School of Medicine, Tokyo, Japan
7. Departmentof Pediatrics,Osaka City University,Osaka, Japan;

Table. List of basal insulin used in the analysis

| Classification | Medication name | Claims code |
| --- | --- | --- |
| Long-acting insulin analog | Insulin Glargine BS Inj.[Lilly] 300 Unit I.U. | 622410901 |
| Long-acting insulin analog | Insulin Glargine BS Injection Kit「FFP」 300 I.U. | 622484801 |
| Long-acting insulin analog | Insulin Glargine BS Inj.[Lilly] 300 I.U. | 622411001 |
| Long-acting insulin analog | Tresiba Injection FlexTouch 300 I.U. | 622198901 |
| Long-acting insulin analog | Tresiba Injection Penfill. 300 I.U. | 622199001 |
| Long-acting insulin analog | LANTUS XR Inj. SoloStar. 450 I.U. | 622440701 |
| Long-acting insulin analog | ANTUS Inj. 100 I.U./mL. | 620008945 |
| Long-acting insulin analog | LANTUS Inj. Cart. 300 I.U. | 620008943 |
| Long-acting insulin analog | LANTUS SoloStar S.C.Injection 300 I.U. | 620007536 |
| Long-acting insulin analog | Levemir InnoLet.300 I.U. | 621927001 |
| Long-acting insulin analog | Levemir FlexPen.300 I.U. | 620008952 |
| Long-acting insulin analog | Levemir Penfill.300 I.U. | 620008953 |
| Intermediate-acting insulin | Novolin N FlexPen. 300 I.U. | 620000266 |
| Intermediate-acting insulin | Humulin N. 100 I.U./mL. | 620008912 |
| Intermediate-acting insulin | Humulin N.300 I.U. | 620008910 |
| Intermediate-acting insulin | Humulin N. 300 I.U. | 622114501 |
| Intermediate-acting insulin | Humalog Injection.　300 I.U. | 620002441 |
| Intermediate-acting insulin | Humalog Injection.　300 I.U. | 620007459 |
| Biphasic insulin | InnoLet 30R.300 I.U. | 640453023 |
| Biphasic insulin | NovoRapid Injection 30 Mix FlexPen. 300 I.U. | 620000448 |
| Biphasic insulin | NovoRapid Injection 30 Mix Penfill. 300 I.U. | 620008896 |
| Biphasic insulin | NovoRapid Injection 50 Mix FlexPen. 300 I.U. | 621973201 |
| Biphasic insulin | NovoRapid Injection 70 Mix FlexPen. 300 I.U. | 621973301 |
| Biphasic insulin | Novolin 30R FlexPen.300 I.U. | 620000269 |
| Biphasic insulin | Humulin 3/7. 100 I.U./mL. | 620008915 |
| Biphasic insulin | Humulin 3/7. 300 I.U. | 620008913 |
| Biphasic insulin | Humulin 3/7. 300 I.U. | 622114601 |
| Biphasic insulin | Humalog Mix25.300 I.U. | 620002439 |
| Biphasic insulin | Humalog Mix25.300 I.U. | 620007461 |
| Biphasic insulin | Humalog Mix50.300 I.U. | 620002440 |
| Biphasic insulin | Humalog Mix50.300 I.U. | 620007462 |
| Biphasic insulin | Ryzodeg combination Injection FlexTouch. 300 I.U. | 622451001 |
| Biphasic insulin | Ryzodeg combination Injection Penfill. 300 I.U. | 622450901 |
